# Incarceration and Quality of Cancer Care

**DOI:** 10.1001/jamanetworkopen.2025.37400

**Published:** 2025-10-14

**Authors:** Oluwadamilola T. Oladeru, Ilana B. Richman, Jenerius A. Aminawung, Jason Weinstein, Lisa B. Puglisi, Rajni Mehta, Hsiu-Ju Lin, Emily A. Wang, Cary P. Gross

**Affiliations:** 1 Department of Radiation Oncology, Mayo Clinic, Jacksonville, Florida; 2 SEICHE Center for Health and Justice, Yale School of Medicine, New Haven, Connecticut; 3 Department of Internal Medicine, Yale School of Medicine, New Haven, Connecticut; 4 Department of Internal Medicine, Yale Cancer Outcomes, Public Policy, and Effectiveness Research (COPPER) Center, New Haven, Connecticut; 5 Yale School of Medicine, New Haven, Connecticut; 6 Yale Cancer Center, Yale School of Medicine, New Haven, Connecticut; 7 University of Connecticut School of Social Work, Hartford

## Abstract

**Importance:**

Although incarceration is associated with worse cancer outcomes, little is known about quality of cancer care among people with a history of incarceration.

**Objective:**

To describe quality of cancer care among those diagnosed with cancer during or immediately after incarceration.

**Design, Setting, and Participants:**

This longitudinal cohort study used Connecticut Tumor Registry data linked to Department of Correction rosters from 2005 through 2016. Patients diagnosed with cancer while incarcerated were matched to those diagnosed within 12 months after incarceration and to those with no history of incarceration. Analysis was performed between March 2024 and January 2025.

**Exposures:**

Incarceration status at time of cancer diagnosis.

**Main Outcomes and Measures:**

The main outcomes were treatment initiation within 60 days and receipt of recommended pretreatment and therapeutic care, ascertained from medical records and tumor registries. Logistic regression was used to evaluate odds of treatment initiation by 60 days. The proportion of recommended elements of pretreatment and therapeutic care received was calculated, and group differences were assessed using χ^2^ tests.

**Results:**

The sample included 690 individuals (233 diagnosed during incarceration, 221 diagnosed within 12 months after incarceration, and 236 with no incarceration history). The mean (SD) age was 49.7 (12.1) years, and 611 (88.6%) were male. Compared with those with no history of incarceration, those diagnosed with cancer during incarceration were less likely to initiate treatment within 60 days (169 of 209 [80.9%] vs 131 of 189 [69.3%]; OR, 0.49 [95% CI, 0.31-0.79]). Initiation of treatment within 60 days was not statistically different for those diagnosed after incarceration vs those with no history of incarceration (144 of 187 [77.0%]; OR, 0.81 [95% CI, 0.49-1.34]). Those diagnosed with local and regional cancers during incarceration received 49.2% of recommended treatment-related care (31 of 63 measures), while those diagnosed after incarceration received 45.3% (34 of 75 measures), compared with 66.7% (36 of 54 measures) among those with no history of incarceration (*P* < .001). People diagnosed with local and regional cancers during incarceration received 80.6% of recommended pretreatment evaluation (54 of 67 measures), and those diagnosed after received 96.8% (59 of 68 measures), compared with 78.1% (50 of 64 measures) among those with no history of incarceration (*P* = .01).

**Conclusions and Relevance:**

In this cohort study of people with a history of incarceration, incarceration was associated with lower likelihood of receiving timely, high-quality cancer treatment. Along with other published literature, this work suggests that gaps in quality of care may contribute to observed disparities in outcomes.

## Key Points

**Question:**

How does the quality of cancer care received by people with a history of incarceration compare to quality of care among those who have not been incarcerated?

**Findings:**

Using statewide data from 690 individuals in Connecticut, this cohort study found that those diagnosed with cancer during incarceration were less likely to initiate treatment within 60 days and had lower adherence to some treatment quality indicators compared with those who have not been incarcerated.

**Meaning:**

This study provides insights into mechanisms that may underlie disparities in cancer outcomes among people with a history of incarceration.

## Teaser

This cohort study evaluates the quality of cancer care received by people with a history of incarcaration compared with those who have not been incarcerated in the US.

## Introduction

The incarcerated population in the US is aging, and an estimated 15% of incarcerated adults, or approximately 175 000 individuals, are now 55 years or older.^1^ With this demographic shift, diseases of aging have become more prevalent, and cancer now ranks as the most common cause of death among people who are incarcerated in the US.^2^ Despite the growing prevalence, cancer outcomes among those incarcerated lag behind those with no history of incarceration. Individuals diagnosed with cancer while incarcerated or immediately following release have an approximate 2-fold increase in cancer-related mortality compared with the general population, even after adjusting for stage at diagnosis.^3^

Although reasons for this disparity are likely complex, one potential mediator may be differences in quality of cancer care. Most correctional systems use outside health systems to provide highly specialized care, including oncologic care.^4^ Outsourcing oncologic care may have complex effects on quality. On one hand, patients may be treated at comprehensive cancer centers and other academic centers, which typically provide access to newer treatments and have been shown to have better outcomes than other cancer treatment settings.^5^ On the other hand, outsourcing care creates additional challenges to delivering high-quality care. These complexities include the logistics of scheduling appointments, arranging transport to outside facilities, communicating across health systems, and sharing medical information. Further, carceral health care is typically financed directly by each state rather than an insurer. Limited budgets may constrain contracts, limit formulary medications, and shape other aspects of care that contribute to quality. These complexities underscore the unique challenges in delivering comprehensive, guideline-concordant cancer care within the constraints of the correctional system.

Research on the quality of cancer care during and after incarceration remains scant. Existing studies have primarily focused on palliative care within prisons or the treatment of specific cancers, such as cervical cancer or breast cancer, or were performed in international settings.^6,7,8,9,10^ These studies suggest significant barriers to timely and accessible cancer treatment, such as neoadjuvant treatments and surgical interventions for incarcerated individuals. However, comparative analyses of cancer care quality between correctional and community health systems across different stages of incarceration—from during incarceration to post-release periods—have not been adequately explored.

The goal of this study was to evaluate the quality of cancer care received by people diagnosed with cancer during and immediately after incarceration. We compared the quality of cancer care among 3 distinct groups: individuals diagnosed with cancer while incarcerated, those diagnosed within 12 months after release, and those never incarcerated. We hypothesized that cancer diagnosis during or shortly after incarceration would be associated with delays in initiating treatment and lower likelihood of receiving recommended care.

## Methods

### Study Design

This longitudinal cohort study used linked administrative data from the Connecticut Tumor Registry and the Connecticut Department of Correction (CT DOC). The Connecticut DOC is an integrated correctional system in which prisons and jails are managed under a single state entity. This integrated administrative structure allows for the identification of persons diagnosed with cancer in prisons and jails statewide by linking DOC rosters to the statewide cancer registry. Our linkage procedures have been previously described.^3,11^ Within this dataset, we identified individuals diagnosed with invasive cancer in Connecticut from 2005 through 2016. We categorized individuals in this group as diagnosed with cancer during incarceration, diagnosed within 12 months after release from incarceration, or not incarcerated during the study window (2005-2016), whom we refer to throughout as “never incarcerated.” Categorization was based on the date of cancer diagnosis reported to the tumor registry and their contact, if any, with the CT DOC during the study period. In cases where an individual was diagnosed with more than 1 invasive cancer, we selected the most advanced cancer for analysis.

The study was approved by the Yale University Institutional Review Board, the Connecticut Department of Public Health Human Investigations Committee, and the CT DOC Research Advisory Council; the need for informed consent was waived because of the impracticality of obtaining consent for thousands of individuals in the cancer registry, and the study was considered non–human participant research. We followed the Strengthening the Reporting of Observational Studies in Epidemiology (STROBE) reporting guideline.

### Study Sample and Data Sources

We matched all individuals diagnosed with invasive cancer during incarceration with those diagnosed within 12 months after release and with those who were never incarcerated for the period between 2005 and 2016. We employed propensity score matching to construct comparable control groups for the analysis. Propensity scores used logistic regression and included the following covariates: age, sex, race and ethnicity, cancer type (eTable 1 in Supplement 1), and stage. Race and ethnicity were obtained from the Connecticut Tumor Registry, which abstracts this information from patient medical records. We incorporated race and ethnicity into this study because they are important proxies of exposure to racism and therefore can be associated with access to care. After estimating the propensity scores, we applied a greedy matching algorithm to match those diagnosed with cancer during incarceration (considered cases) to those diagnosed after incarceration and those with no history of incarceration (considered controls). Where more than 1 match was identified, a matched control was selected randomly. We chose a 1:1 case-control match method, rather than inverse weighting or stratification, because it produced the most balanced distribution of covariates for our sample. The 1:1 case-control method often outperforms other matching methods by minimizing model misspecification, especially when the sample size for a comparison group is much larger than the treatment group.

For analyses evaluating receipt of recommended care, we included patients diagnosed with 1 of 7 common cancer types: breast, cervical, colorectal, hepatocellular, lung, melanoma, and prostate. We chose these cancer types because quality of care is well defined in the literature (eTable 2 in Supplement 1). We excluded patients with metastatic disease at diagnosis. In sensitivity analysis, we included patients who had metastatic disease at the time of diagnosis.

To ascertain details of cancer diagnosis and treatment, we abstracted medical records from 2 academic medical centers where patients received cancer care and from the statewide cancer registry. Records from all 3 sources were reviewed for each case to identify all relevant information. Using a standardized abstraction tool, we captured detailed information about cancer care received by participants, including dates and types of diagnostic procedures, pretreatment testing, and treatments administered. Experienced medical record abstractors from the Yale Cancer Rapid Case Ascertainment Shared Resource, which has a collaborative partnership with the Connecticut Tumor Registry, conducted the medical record abstractions using a standardized approach.

### Measures

The primary independent variable was incarceration status at the time of cancer diagnosis, categorized into 3 groups: incarcerated, post release, or never incarcerated. If incarceration status changed during the period of treatment, the individual was assigned to the setting where diagnosis occurred. The primary outcome measures were time to treatment initiation, including surgery, chemotherapy, and radiation therapy, and receipt of recommended cancer care. For time to treatment analyses, we defined time to treatment as the time from the date of diagnosis, as recorded in the Connecticut Tumor Registry, to the date the participant received their initial cancer-directed treatment. We also specifically examined whether treatment was initiated within 60 days following the diagnosis for all cancer types included in our study as a binary variable.^12^

To define receipt of recommended care, we used published quality measures (eTable 2 in Supplement 1) focusing on pretreatment evaluation and treatment, as these domains are most closely associated with cancer outcomes. We chose quality measures that reflect evidence-based practice and that were endorsed by the National Quality Forum. Using these measures, we identified every element of recommended care that each individual was eligible for, based on the individual’s cancer type and stage at diagnosis. We then assessed whether the individual received each element of recommended care, based on data abstracted from medical records and the statewide cancer registry.

We then calculated a quality measure score, aggregated at the group level by place of cancer diagnosis (ie, diagnosed during incarceration, after incarceration, or in the community/never incarcerated). The quality measure score was calculated as the number of elements of recommended care received in the population divided by the total number of eligible elements of care in the population. Each individual in the sample could contribute multiple quality measures, depending on the cancer type and stage. Our sample included patients diagnosed with cancer over a 10-year period during which diagnostic standards and treatment patterns changed. Therefore, when assessing quality of care, we did not anticipate complete adherence and focused analysis on comparison of quality between cohorts.

### Statistical Analysis

Analysis was conducted between March 2024 and January 2025. We compared demographic and cancer characteristics in the matched samples using χ^2^ or Fisher exact tests. For time-to-treatment analyses, we compared the median time to treatment initiation by incarceration status at diagnosis using the Wilcoxon test. We evaluated time to treatment among all patients with cancer and among the subgroup with nonmetastatic disease at baseline. In a sensitivity analysis, we excluded individuals for whom diagnosis and treatment occurred on the same day (for example, by surgical excision at the time of diagnosis).

We also examined receipt of initial cancer treatment within 60 days as a binary outcome among all 3 groups. Our analysis compared receipt of treatment within 60 days using simple proportions and logistic regression. Logistic models excluded people who died within 60 days of diagnosis. We evaluated unadjusted models as well as models adjusted for age, sex, race and ethnicity, and cancer stage at diagnosis to account for any residual differences after matching. In sensitivity analysis, we excluded patients with metastatic disease at diagnosis.

Our analysis of quality measure scores included all patients diagnosed with 1 of 7 common cancers. We compared quality measure scores for pretreatment measures, treatment measures, and overall across all 3 groups using χ^2^ tests.^13^ In our primary analysis, we compared receipt of recommended cancer care among patients with nonmetastatic cancer at diagnosis, as treatment is often more variable when cancer is metastatic at diagnosis. We weighted the analysis to account for multiple quality measures per case. In a sensitivity analysis, we included all cancer stages (including those diagnosed at a metastatic stage). To maintain the confidentiality of individual records, we only reported results that included at least 11 unweighted cases in any cell.^14^ Additionally, data in some categories were coarsened to maintain privacy. A 2-sided *P* < .05 was considered statistically significant. We conducted all analyses in SPSS, version 26 (IBM Inc).

## Results

Our final matched cohort included 690 individuals, of whom 233 (33.8%) were diagnosed with cancer during incarceration, 221 (32.0%) were diagnosed with cancer within 12 months after incarceration, and 236 (34.2%) had no incarceration history. The groups were successfully matched, with a mean (SD) age of 49.7 (12.1) years. A total of 611 (88.6%) were male and 78 (11.3%) were female. A total of 118 (17.1%) were identified as Hispanic, 228 (33.0%) as non-Hispanic Black, more than 326 (>47.2%) as non-Hispanic White, and the remainder (<11) as other race or ethnicity in the tumor registry data (Table 1). Among the never-incarcerated group, 96 (40.7%) had private insurance, compared with 20 (9.0%) in the postrelease group. Among those in the postrelease group, most had Medicaid insurance (131 [59.3%]), and for the incarcerated group, the CT DOC financed health care costs. In the matched cohort, 310 individuals overall (44.9%) were diagnosed with either breast, cervical, colorectal, hepatocellular, lung, melanoma, or prostate cancer, which were included in analyses of recommended care.

**Table 1.** Characteristics of Study Sample by Incarceration Status at Cancer Diagnosis^a^

Characteristic	Total No. (N = 690)	No. (%)			*P* value
		Never incarcerated (n = 236)	Incarcerated (n = 233)	Post release (n = 221)	
Age, mean (SD) [range], y	49.77 (12.09) [18-88]	49.70 (12.92) [18-88]	49.39 (12.49) [18-88]	50.23 (10.71) [21-78]	.75
Age group, y					
<35	81	32 (13.6)	30 (12.9)	19 (8.6)	.46
35-49	246	79 (33.5)	82 (35.2)	85 (38.5)	
≥50	363	125 (53.0)	121 (51.9)	117 (52.9)	
Sex^b^					
Male	611	214 (90.7)	208 (89.7)	189 (85.5)	.19
Female	78	22 (9.3)	24 (10.3)	32 (14.5)	
Race and ethnicity					
Hispanic	118	42 (17.8)	38 (16.4)	38 (17.2)	.96
Non-Hispanic Black	228	77 (32.6)	83 (35.8)	68 (30.8)	
Non-Hispanic White^c^	>326	>105 (44.4)	>103 (44.2)	>106 (>48.0)	
Non-Hispanic other^a^^,^^d^	<11	<11 (<5)	<11 (<5)	<11 (<5)	
Primary insurance at diagnosis					
DOC, no insurance, or unknown	334	63 (26.7)	233 (100)	38 (17.1)	<.001
Medicaid	173	42 (17.8)	0	131 (59.3)	
Medicare	67	35 (14.8)	0	32 (14.5)	
Private	116	96 (40.7)	0	20 (9.0)	
Cancer stage at diagnosis					
Localized	197	64 (27.1)	66 (28.4)	67 (30.3)	.84
Regional	163	56 (23.7)	56 (24.1)	51 (23.1)	
Distant^c^	306	>108 (>45.8)	>102 (>43.8)	>87 (39.4)	
Unknown	23	<11 (<5)	<11 (<5)	<11 (<5)	
Primary tumor					
Breast	14	<11 (<5)	<11 (<5)	<11 (<5)	.03
Cervix	<11	<11 (<5)	<11 (<5)	<11 (<5)	
Colon	67	30 (12.7)	23 (9.9)	14 (6.3)	
Liver^c^	>42	<11 (<5)	14 (7.3)	26 (11.8)	
Lung	108	42 (17.8)	38 (16.3)	28 (12.7)	
Melanoma	<11	<11 (<5)	<11 (<5)	<11 (<5)	
Prostate	58	21 (8.9)	18 (7.7)	19 (8.6)	
Other	379	131 (55.5)	126 (54.1)	122 (55.2)	

Abbreviation: DOC, Department of Correction.

^a^ Cell sizes containing fewer than 11 individuals are suppressed due to privacy concerns.

^b^ Sex was missing for 1 individual.

^c^ Data coarsened to maintain privacy.

^d^ Other race and ethnicity included Asian or Pacific Islander and American Indian or Alaska Native.

In the incarcerated group, the median time to treatment was 33 days (IQR, 0-70 days) for those with local and regional stage at diagnosis, compared with 31 days in the postrelease group (IQR, 0.50-60 days) and 21 days in the never incarcerated group (IQR, 0-40 days) (*P* = .01). Results were similar after excluding patients treated on the day of diagnosis. For all tumor stages, 131 of 189 of those diagnosed during incarceration (69.3%) initiated treatment within 60 days, compared with 144 of 187 (77.0%) and 169 of 209 (80.9%) in the postrelease and never incarcerated groups, respectively (*P* = .02) (Table 2). In adjusted analyses, odds of treatment at 60 days were lower among those diagnosed during incarceration compared with those who had never been incarcerated (odds ratio [OR], 0.49; 95% CI, 0.30-0.79) but not among those diagnosed post release (OR, 0.81; 95% CI, 0.49-1.34) (Table 3). Results were similar when analyses were limited to those with nonmetastatic disease at diagnosis.

**Table 2.** Time to Cancer Treatment Initiation by Place of Diagnosis in Days

Cancer stage	Never incarcerated	Incarcerated	Post release	*P* value
**Local and regional tumors**				
Total No.	118	109	108	NA
Days to treatment initiation, median (IQR)	21 (0-40)^a^^,^^b^	33 (0-70)^a^	31 (0.50-60)^b^	.01
Days to treatment initiation (excluding same day), median (IQR)	32 (25-62)^a^	49 (31-88)^a^	42 (28-72)	.006
**All stages**				
Total No.	209	189	187	NA
Days to treatment initiation, median (IQR)	22 (0-39.5)	30 (0.75-58.25)	28 (3-54)	.05
Days to treatment initiation (excluding same day), median (IQR)	31 (19-57)	40 (21-70)	34 (15-60)	.11
Treatment initiated within 60 d, No. (%)	169 (80.9)^a^	131 (69.3)^a^	144 (77.0)	.02

Abbreviation: NA, not applicable.

^a^ Significant difference between never incarcerated and incarcerated groups.

^b^ Significant difference between never incarcerated and postrelease groups.

**Table 3.** Receipt of Initial Cancer Treatment Within 60 Days Among Patients Who Survived 60 Days or More

Variable	Unadjusted odds ratio (95% CI)	*P* value	Adjusted odds ratio (95% CI)	*P* value
Incarceration status at diagnosis (reference = never incarcerated)				
Incarcerated	0.53 (0.34-0.85)	.01	0.49 (0.30-0.79)	.004
Post release	0.79 (0.49-1.29)	.35	0.81 (0.49-1.34)	.40
Age, years	NA	NA	0.95 (0.94-0.97)	<.001
Sex (reference = female)	NA	NA	1.08 (0.58-2.01)	.81
Race (reference = non-Hispanic White)				
Non-Hispanic Black	NA	NA	0.96 (0.62-1.49)	.84
Hispanic	NA	NA	1.14 (0.65-2.02)	.65
Cancer SEER stage at diagnosis (reference = localized)				
Regional	NA	NA	2.08 (1.21-3.59)	.01
Distant	NA	NA	1.45 (0.92-2.28)	.10

Abbreviations: NA, not applicable SEER, Surveillance, Epidemiology, and End Results.

Among the 310 individuals diagnosed with 1 of 7 cancer types for whom quality of care was assessed, 185 (59.7%) were diagnosed at nonmetastatic stage. Overall, we did not observe statistically significant differences in the quality measure scores based on place of diagnosis. Among people with no history of incarceration, 85 of 118 (72.0%) quality measures overall were met, compared with 83 of 130 (63.8%) among the incarcerated group and 94 of 143 (65.7%) among the postrelease group (*P* = .33) (Table 4). However, we did observe differences among treatment and pretreatment quality measures by place of diagnosis. For treatment-related quality measures, among those with no history of incarceration, 36 of 54 quality measures (66.7%) were met, whereas 31 of 63 measures (49.2%) were met among those diagnosed while incarcerated and 34 of 75 measures (45.3%) were met among those diagnosed post release (*P* < .001). For pretreatment measures, quality of care scores were not statistically different among those with no history of incarceration (50 of 64 measures met [78.1%]) compared with those diagnosed during incarceration (54 of 67 measures met [80.6%]) and were higher among those diagnosed post incarceration (59 of 68 measures met [86.8%]) compared with those with no history of incarceration (Table 4). Results were similar when including people with metastatic cancer at the time of diagnosis.

**Table 4.** Quality of Cancer Care for 7 Common Cancers by Place of Cancer Diagnosis

	Measures		Total measures met, No./total No. (%)			*P* value
	No.	No. of recommended care elements received/total No. of possible elements (%)^a^	Never incarcerated	Incarcerated	Post release	
**Local and regional cancers**						
No. of individuals	NA		59	59	67	NA
Domain of care						
Pretreatment	9	164/199 (82.4)	50/64 (78.1)^b^	54/67 (80.5)	59/68 (86.8)^b^	.01
Treatment	16	102/192 (53.1)	36/54 (66.7)^c^^,^^b^	31/63 (49.2)^c^	34/75 (45.3)^b^	<.001
Total eligible events	24	262/391 (67.0)	85/118 (72.0)	83/130 (63.8)	94/143 (65.7)	.33
Total assessed, No.	38	1084	352	340	392	NA
**All cancers**						
No. of individuals	NA		105	106	99	NA
Domain of care						
Pretreatment	9	229/350 (65.4)	79/127 (62.2)	79/121 (65.3)	70/102 (68.6)	.54
Treatment	16	213/372 (57.3)	86/132 (65.1)^b^	67/124 (54.0)	61/116 (52.6)^b^	.02
Total eligible events	24	453/722 (62.7)	169/259 (65.3)	150/245 (61.2)	135/218 (61.9)	.15
Total assessed, No.	38	1979	685	674	620	NA

Abbreviation: NA, not applicable.

^a^ Values are weighted to account for the fact that individuals were eligible for multiple quality measures.

^b^ Significant difference between never incarcerated and postrelease groups.

^c^ Significant difference between never incarcerated and incarcerated groups.

## Discussion

In this study of quality of cancer care, we found that time to treatment was generally longer for those diagnosed with cancer during incarceration compared with those with no history of incarceration. We also found lower rates of adherence to treatment-related quality measures among those diagnosed during and after incarceration. Conversely, the quality of care related to pretreatment evaluation was similar or better among those with a history of incarceration compared with those never incarcerated.

The unique structures of incarceration shape many dimensions of cancer care, including diagnosis, decision-making, access to care, sharing of medical records, and trustworthiness of medical care. A recent set of studies examined cancer care in the UK and found that quality of care was worse among those diagnosed during incarceration compared with those not incarcated.^10^ Experiences of care were influenced by incarceration itself, including distrust, stigmatization, and poor physician-patient communication.^10^ The UK provides a particularly instructive case, as health care in prisons and jails is delivered through the National Health Service, as it is for the general public, and clinicians practicing in carceral settings are expected to follow similar standards. Thus, disparities in care reflect the effects of incarceration itself, including the ways in which carceral systems erode trust, limit access to health information, and reduce availably of family and community support.

In the US, systems of care are more variable and often include a hybrid model in which primary care is provided by departments of corrections (or their contractors), while specialist care is provided under contract with community or academic clinicians.^15^ This hybrid model creates additional challenges in coordination of care that can impact complex specialty care for people with cancer during incarceration. Limited availability of appointments, inadequate information from specialists, and logistical challenges around care coordination can impact timely and efficient care delivery.

These factors continue to shape care during the postincarceration period where lack of continuity of care, disruptions in health insurance (which is typically suspended or terminated during incarceration), linking of health insurance to employment, and health system complexity may contribute to worse outcomes.^16,17,18^ Indeed, disruptions in health insurance are associated with delays in time to treatment and higher mortality in the general population, while insurance coverage expansions have been associated with shorter times to treatment and reduced disparities in cancer care delivery.^19,20^ Our findings of longer time to treatment and lower treatment quality scores in the recently incarcerated population support the notion that structural determinants of health, such as insurance coverage, are likely to influence quality of care even after incarceration.

How might disparities in time to treatment and quality of care be addressed? First, greater transparency about quality of care and cancer outcomes in correctional facilities could help identify opportunities for improvement and create accountability. Recent federal legislation that requires strengthened oversight for health care and safety in federal facilities provides a useful first step for improving transparency, accountability, and quality and may serve as a model for similar state-based standards.^21,22,23^ Community clinicians and specialists, including at academic centers and cancer centers, must also work to improve care for people during and after incarceration. Interventions that have been successful in the general population for improving quality and timeliness of care, including patient navigation, interoperability of medical records, and provision of coordinated, team-based care, may also help reduce disparities for those diagnosed with cancer during incarceration.^24,25,26^

In the postincarceration period, community clinicians must also be attentive to the particular needs of this population, including limited health literacy, insurance gaps, and logistical barriers such as transportation.^16^ Additional supports, such as community health workers with lived experience of incarceration, may bridge gaps in health literacy and assist in navigating health systems and have been shown to reduce emergency department use post incarceration.^27^ Cancer centers and health systems must leverage these strategies to ensure high-quality, timely care for people who are currently or formerly incarcerated. Lastly, policy-level interventions, including ensuring health insurance coverage during and after incarceration, can prevent delays and disruptions in care and could reduce disparities in timely diagnosis and treatment.^19^

### Limitations

This study has important limitations. First, we used data from a single state, Connecticut. All states organize carceral health care independently, and findings from Connecticut may not apply to all states. Still, most states share common features with Connecticut, including contracting with community organizations to provide cancer care. Second, data used in this study are from 2005 through 2016, which may limit relevance to current practice, though to our knowledge, quality of cancer care has not been a focus of coordinated quality improvement efforts broadly. Third, we examined receipt of recommended care among a limited number of common cancer types. We chose this strategy because quality of care is less well defined for more rare cancers. However, because we evaluated receipt of recommended care in a subset of patients, we had limited power to detect smaller differences in quality of care between groups. Fourth, other social determinants of health, including health literacy, educational attainment, and social support may contribute to the findings, as these factors may differ between people with a history of incarceration and those never incarcerated, and future work could focus on understanding the interplay between social determinants of heath, incarceration, and cancer outcomes. Additionally, transitions of care may have important effects on quality of care and outcomes. Although we did not explore transitions in this study, future work could evaluate how discharge or reincarceration during a cancer diagnosis impacts quality and outcomes. Lastly, we focused on 3 main domains of quality: timeliness of treatment, pretreatment evaluation, and receipt of recommended treatments. We were unable to evaluate other important domains of quality, including patient experience, given data availability.

## Conclusions

In this cohort study from the state of Connecticut, we observed that some measures of quality of care differed among people diagnosed with cancer during incarceration compared with those who were not incarcerated. In particular, time to treatment was longer among those diagnosed while incarcerated, while receipt of recommended care did not differ overall. Previous research has shown that people diagnosed with cancer during incarceration have worse health outcomes, and our findings provide initial evidence of areas where quality improvement efforts might begin. Carceral settings might consider adapting interventions shown to be effective in the community to the carceral environment, including continuous quality measurements on factors related to time to initiation of treatment and use of patient navigation and care coordination, to understand and address disparities in cancer care. Community clinicians may also consider identifying supports to promote continuity of care for patients with a history of legal system involvement. Such efforts may help reduce disparities in cancer care for patients diagnosed during and after incarceration.

## Notes

Supplement 1. **eTable 1. **List of cancer types diagnosed and categorized into cancer groups **eTable 2.** Quality measures by cancer site

Supplement 2.
Data Sharing Statement
