# Supplementary material for: Incarceration and Quality of Cancer Care
Source: JAMA Netw Open. 2025 Oct 14;8(10):e2537400. doi: 10.1001/jamanetworkopen.2025.37400 (PMC12522002; doi:10.1001/jamanetworkopen.2025.37400)
Supplement: Supplement 1. — eTable 1. List of cancer types diagnosed and categorized into cancer groups eTable 2. Quality measures by cancer site [file jamanetwopen-e2537400-s001.pdf]

Supplemental Online Content

Oladeru OT, Richman IB, Aminawung JA, et al. Incarceration and quality of cancer care. *JAMA Netw Open*. 2025;8(10):e2537400. doi:10.1001/jamanetworkopen.2025.37400

eTable 1. List of cancer types diagnosed and categorized into cancer groups

eTable 2. Quality measures by cancer site

This supplemental material has been provided by the authors to give readers additional information about their work.

© 2025 Oladeru OT et al. *JAMA Network Open*.

<u>eTable 1:</u> List of cancer types diagnosed and categorized into cancer groups

ICD code	Site Group	Cancer Group
C000-C009	Lip	Head and Neck
C019-C029	Tongue	Head and Neck
C079-C089	Salivary Gland	Head and Neck
C040-C049	Floor of Mouth	Head and Neck
C030-C039, C050-C059, C060-C069	Gum and Other Mouth	Head and Neck
C110-C119	Nasopharynx	Head and Neck
C090-C099	Tonsil	Head and Neck
C100-C109	Oropharynx	Head and Neck
C129, C130-C139	Hypopharynx	Head and Neck
C140, C142, C148	Other Oral Cavity and Pharynx	Head and Neck
C150-C159	Esophagus	Gastrointestinal
C160-C169	Stomach	Gastrointestinal
C170-C179	Small Intestine	Gastrointestinal
C180	Cecum	Gastrointestinal
C181	Appendix	Gastrointestinal
C182	Ascending Colon	Gastrointestinal
C183	Hepatic Flexure	Gastrointestinal
C184	Transverse Colon	Gastrointestinal
C185	Splenic Flexure	Gastrointestinal
C186	Descending Colon	Gastrointestinal
C187	Sigmoid Colon	Gastrointestinal
C188-C189, C260	Large Intestine, NOS	Gastrointestinal
C199	Rectosigmoid Junction	Gastrointestinal

© 2025 Oladeru OT et al. *JAMA Network Open*.

C209	Rectum	Gastrointestinal
C210-C212, C218	Anus, Anal Canal and Anorectum	Gastrointestinal
C220	Liver	Gastrointestinal
C221	Intrahepatic Bile Duct	Gastrointestinal
C239	Gallbladder	Gastrointestinal
C240-C249	Other Biliary	Gastrointestinal
C250-C259	Pancreas	Gastrointestinal
C480	Retroperitoneum	Gastrointestinal
C481-C482	Peritoneum, Omentum and Mesentery	Gastrointestinal
C268-C269, C488	Other Digestive Organs	Gastrointestinal
C300-C301, C310-C319	Nose, Nasal Cavity and Middle Ear	Head and Neck
C320-C329	Larynx	Head and Neck
C340-C349	Lung and Bronchus	Thoracic
C384	Pleura	Thoracic
C339, C381-C383, C388, C390, C398, C399	Trachea, Mediastinum and Other Respiratory Organs	Thoracic
C400-C419	Bones and Joints	Sarcoma
C380, C470-C479, C490-C499	Soft Tissue including Heart	Sarcoma
C440-C449	Melanoma of the Skin	Skin
C440-C449	Other Non-Epithelial Skin	Skin
C500-C509	Breast	Breast
C530-C539	Cervix Uteri	Female Reproductive (gynecology)
C540-C549	Corpus Uteri	Female Reproductive (gynecology)
C559	Uterus, NOS	Female Reproductive (gynecology)
C569	Ovary	Female Reproductive (gynecology)
C529	Vagina	Female Reproductive (gynecology)
C510-C519	Vulva	Female Reproductive (gynecology)
C570-C579, C589	Other Female Genital Organs	Female Reproductive (gynecology)
C619	Prostate	Male Reproductive

© 2025 Oladeru OT et al. *JAMA Network Open*.

C620-C629	Testis	Male Reproductive
C600-C609	Penis	Male Reproductive
C630-C639	Other Male Genital Organs	Male Reproductive
C670-C679	Urinary Bladder	Urinary (non-reproductive)
C649, C659	Kidney and Renal Pelvis	Urinary (non-reproductive)
C669	Ureter	Urinary (non-reproductive)
C680-C689	Other Urinary Organs	Urinary (non-reproductive)
C690-C699	Eye and Orbit	Central Nervous System (CNS)
C710-C719	Brain	Central Nervous System (CNS)
C710-C719	Cranial Nerves Other Nervous System	Central Nervous System (CNS)
C739	Thyroid	Head and Neck
C379, C740-C749, C750-C759	Other Endocrine including Thymus	Head and Neck
C024, C098-C099, C111, C142, C379, C422, C770-C779	Hodgkin - Nodal	Leukemia & Lymphoma
All other sites	Hodgkin - Extranodal	Leukemia & Lymphoma
C024, C098, C099, C111, C142, C379, C422, C770-C779	NHL - Nodal	Leukemia & Lymphoma
All sites except C024, C098-C099, C111, C142, C379, C422, C770-C779	NHL - Extranodal	Leukemia & Lymphoma
	Myeloma	Leukemia & Lymphoma
	Acute Lymphocytic Leukemia	Leukemia & Lymphoma
C420, C421, C424	Chronic Lymphocytic Leukemia	Leukemia & Lymphoma
	Other Lymphocytic Leukemia	Leukemia & Lymphoma
	Acute Myeloid Leukemia	Leukemia & Lymphoma
	Chronic Myeloid Leukemia	Leukemia & Lymphoma
	Other Myeloid/Monocytic Leukemia	Leukemia & Lymphoma
	Acute Monocytic Leukemia	Leukemia & Lymphoma
	Other Acute Leukemia	Leukemia & Lymphoma
	Aleukemic, subleukemic and NOS	Leukemia & Lymphoma

© 2025 Oladeru OT et al. *JAMA Network Open*.

	Mesothelioma	Thoracic
	Kaposi Sarcoma	Sarcoma
	Miscellaneous	Other

© 2025 Oladeru OT et al. *JAMA Network Open*.

eTable 2: Quality measures by cancer site

Site	Measure	*Source*
**BREAST**		
Pre-treatment	Use of Needle biopsy before BCS/mastectomy	*ASCOand ACS Screening guidelines*
	If needle biopsy or surgery, then ER, PR and HER2	*ASCO and ACS Screening guidelines*
Treatment	If age<70, and BCS, THEN radiation therapy within 365 days of dx	*NQF 0219*
	If BCS or mastectomy and >=4 positive regional nodes, THEN radiation therapy within 365 days of dx	*NQF 0219*
	If stage I to III and ER/PR positive, THEN Tamoxifen/Aromatase initiation within 1year of diagnosis	*NQF 0220*
	If age<70, stage I to III and HR negative, THEN chemotherapy within 120 days of diagnosis	*NQF 0559*
	If stage I to III, HER2 positive and adjuvant chemo, THEN Trastuzumab	*NQF 1858*
**LUNG**		
Pre-treatment	If NSCLC and chemotherapy, THEN EGFR test before chemo	*Wang X et al. BMC Cancer. 2017;17(1):603*
	If NSCLC and stage III or IV , THEN Brain imaging within 3 months of start of treatment	*Mazzone, P. et al. (2014). Chest, 146(3), 659-669.(https://www.sciencedirect.com/science/article/pii/S0012369215510738?via%3Dihub)*
	If stage >= IB, and no metastasis and Rx intent curative, THEN mediastinal lymph node sampled	*Mazzone, P. et al. (2014). Chest, 146(3), 659-669.*
Treatment	If NSCLC and (stage IIIB and malignant effusion) or stage IV, THEN first line chemo	*Wang X et al. BMC Cancer. 2017;17(1):603; https://pubmed.ncbi.nlm.nih.gov/16384540/*
	If surgery, THEN >=10 lymph nodes removed and >= 3 mediastinal lymph nodes sampled	*Chiew, K.,et al. (2018). Journal of Thoracic Oncology, 13(10), S993.*

© 2025 Oladeru OT et al. *JAMA Network Open*.

	If NSCLC and stage IIIB and surgery, THEN RT at same time as surgery	*ASCO (https://ascopubs.org/doi/full/10.1200/JCO.21.02528)*
	If chemotherapy and Died, THEN chemo < 2wks from death	*Vrijens, F. et al. (2018). International Journal for Quality in Health Care, 30(4), 306-312.*
	If NSCLC and stage IIA, IIB or IIIA and surgery, THEN cisplatin-based chemo within 3 to 4 weeks	*Wang X et al. BMC Cancer. 2017;17(1):603*
	If stage I or II or III and RT and died, THEN death <60 after RT	*Vrijens, F. et al. (2018). International Journal for Quality in Health Care, 30(4), 306-312.*
**PROSTATE**		
Pre-treatment	If PSA, then PSA level at diagnosis recorded	*Ortelli, L., et ak. (2018). BMC cancer, 18(1), 733.*
Treatment	If no mets and (Gleason>=8 or PSA>=20, THEN curative intent surgery or RT	*Ortelli, L., et ak. (2018). BMC cancer, 18(1), 733.*
	If no Mets and (Gleason>=8 or PSA>=20, THEN hormonal therapy before surgery (neoadjuvant HT) and RT	*Ortelli, L., et ak. (2018). BMC cancer, 18(1), 733.*
	If mets, THEN hormonal therapy within 90days of diagnosis	*Ortelli, L., et ak. (2018). BMC cancer, 18(1), 733.*
	If no mets and surgery, THEN death within 30 days of surgery	*Ortelli, L., et ak. (2018). BMC cancer, 18(1), 733.*
	If PSA >20ng/ml or Gleason >=8 or Gleason group >=4 or stage >=III, and external beam RT, THEN androgen deprivation therapy	*NQF 0390*
**CRC**		
Pre-treatment	If surgery, then CEA & CA19-9 before surgery	*Bao, H. et al. (2016). Journal of cancer research and clinical oncology, 142(5), 1079-1089.*
	If surgery, then CT/MRI before surgery	*Bao, H. et al. (2016). Journal of cancer research and clinical oncology, 142(5), 1079-1089.; NQF*
Treatment	If surgery, THEN >= 12 regional nodes examined	*Bao, H. et al. (2016). Journal of cancer research and clinical oncology, 142(5), 1079-1089.*
	If age<80, stage III, and surgery, THEN chemotherapy within 120 days of surgery	*NQF 0223*
**CERVIX**		
Treatment	If radiation therapy, then time to treatment is <= 60days	*CBRRT (https://www.facs.org/media/arldkl5o/quality-measures.pdf)*

© 2025 Oladeru OT et al. *JAMA Network Open*.

	If RT and stages IB -IV, or positive pelvic nodes, margins or parametrium, THEN chemotherapy	*CBRRT*
**MELANOMA**		
Treatment	If inguinal lymph node dissection, THEN >= 5 nodes examined	*M05IgLN (https://www.facs.org/media/arldkl5o/quality-measures.pdf)*
	If axillary lymph node dissection, THEN >= 10 nodes examined	*M10AxLN*
	If positive sentinel lymph node biopsy, THEN Lymph node dissection performed	*MCLND*
	If BRAF mutation test, THEN test before first treatment (surgery/chemo/immunoRx)	*Hawryluk EB, Tsao H. Melanoma: clinical features and genomic insights. Cold Spring Harb Perspect Med. 2014;4(9):a015388. Published 2014 Sep 2. doi:10.1101/cshperspect.a015388*
	If BRAF positive, then immunotherapy with BRAF inhibitors (vemurafenib and dabrafenib)	*Hertzman Johansson C, Egyhazi Brage S. BRAF inhibitors in cancer therapy. Pharmacol Ther. 2014;142(2):176-182. doi:10.1016/j.pharmthera.2013.11.011*
**HCC**		
Pre-treatment	If cirrhosis and tumor size > 2cm, THEN diagnosis method = imaging (US, CT or MRI) or imaging and biopsy	*EASL-EORTC Clinical Practice Guidelines (CPG) 2012 - https://www.ejcancer.com/article/S0959-8049(11)01070-7/fulltext*
Treatment	If nodular tumor and tumors=1 (and preserved liver function), THEN surgical resection is performed	*EASL-EORTC Clinical Practice Guidelines (CPG) 2012 - https://www.ejcancer.com/article/S0959-8049(11)01070-7/fulltext*
	If BCLC stage A, THEN radiofrequency ablation or ethanol injection and NO surgery	*EASL-EORTC Clinical Practice Guidelines (CPG) 2012 - https://www.ejcancer.com/article/S0959-8049(11)01070-7/fulltext*
	If BCLC stage B and nodular (>1 tumor), THEN chemoembolization	*EASL-EORTC Clinical Practice Guidelines (CPG) 2012 - https://www.ejcancer.com/article/S0959-8049(11)01070-7/fulltext*
	If BCLC stage D, then palliative care and no clinical trial participation	*EASL-EORTC Clinical Practice Guidelines (CPG) 2012 - https://www.ejcancer.com/article/S0959-8049(11)01070-7/fulltext*

© 2025 Oladeru OT et al. *JAMA Network Open*.
