# Supplementary material for: Incarceration and Quality of Cancer Care
Source: JAMA Netw Open. 2025 Oct 14;8(10):e2537400. doi: 10.1001/jamanetworkopen.2025.37400 (PMC12522002; doi:10.1001/jamanetworkopen.2025.37400)
Supplement: Supplement 2. — Data Sharing Statement [file jamanetwopen-e2537400-s002.pdf]

Data Sharing Statement

Oladeru. Incarceration and Quality of Cancer Care. *JAMA Netw Open*. Published October 14, 2025. doi:10.1001/jamanetworkopen.2025.37400

Data
Data available: No

Additional Information
Explanation for why data not available: We are not permitted to share patient level data under our current DUA.
